# Ubiquitin ligase RNF123 Mediates Degradation of Heterochromatin Protein 1α and β in Lamin A/C Knock-Down Cells

**DOI:** 10.1371/journal.pone.0047558

**Published:** 2012-10-15

**Authors:** Pankaj Chaturvedi, Richa Khanna, Veena K. Parnaik

**Affiliations:** Centre for Cellular and Molecular Biology (CSIR), Hyderabad, India; Mayo Clinic, United States of America

## Abstract

**Background:**

The nuclear lamina is a key determinant of nuclear architecture, integrity and functionality in metazoan nuclei. Mutations in the human lamin A gene lead to highly debilitating genetic diseases termed as laminopathies. Expression of lamin A mutations or reduction in levels of endogenous A-type lamins leads to nuclear defects such as abnormal nuclear morphology and disorganization of heterochromatin. This is accompanied by increased proteasomal degradation of certain nuclear proteins such as emerin, nesprin-1α, retinoblastoma protein and heterochromatin protein 1 (HP1). However, the pathways of proteasomal degradation have not been well characterized.

**Methodology/Principal Findings:**

To investigate the mechanisms underlying the degradation of HP1 proteins upon lamin misexpression, we analyzed the effects of shRNA-mediated knock-down of lamins A and C in HeLa cells. Cells with reduced levels of expression of lamins A and C exhibited proteasomal degradation of HP1α and HP1β but not HP1γ. Since specific ubiquitin ligases are upregulated in lamin A/C knock-down cells, further studies were carried out with one of these ligases, RNF123, which has a putative HP1-binding motif. Ectopic expression of GFP-tagged RNF123 directly resulted in degradation of HP1α and HP1β. Mutational analysis showed that the canonical HP1-binding pentapeptide motif PXVXL in the N-terminus of RNF123 was required for binding to HP1 proteins and targeting them for degradation. The role of endogenous RNF123 in the degradation of HP1 isoforms was confirmed by RNF123 RNAi experiments. Furthermore, FRAP analysis suggested that HP1β was displaced from chromatin in laminopathic cells.

**Conclusions/Significance:**

Our data support a role for RNF123 ubiquitin ligase in the degradation of HP1α and HP1β upon lamin A/C knock-down. Hence lamin misexpression can cause degradation of mislocalized proteins involved in key nuclear processes by induction of specific components of the ubiquitin-proteasome system.

## Introduction

Lamins are intermediate filament proteins that form a filamentous network underlying the inner nuclear membrane termed the nuclear lamina. Most of the higher vertebrates contain two types of lamins, classified primarily on basis of expression patterns and biochemical properties: A-type and B-type lamins. The A-type lamins (A and C) are alternatively spliced products of the lamin A gene that are expressed in differentiated cells, whereas the B-type lamins (B1 and B2) are encoded by two separate genes and are expressed in all somatic cells. Lamins play important roles in the organization of nuclear functions such as DNA replication and transcription, as well as in chromatin organization and gene regulation. Mutations in the human lamin A gene (*LMNA*) lead to at least 15 debilitating genetic diseases, collectively referred to as laminopathies. These include muscular dystrophies, cardiomyopathies, lipodystrophies and progerias [1]–[5].

At the cellular level, mutations in lamin A/C or decrease in lamin A/C levels cause abnormal nuclear morphology and loss in heterochromatic markers, which are accompanied by impaired nuclear function. Downregulation of the single lamin gene of *C. elegans* causes abnormal heterochromatin organization leading to embryonic lethality [6], while misexpression or reduction of *Drosophila* A-type lamin results in disruption of heterochromatin protein 1 (HP1) localization [7]. Similarly, fragmentation of heterochromatin has been observed in cardiomyocytes derived from *lmna* ^−/−^ mice [8]. Furthermore, heterochromatin markers such as trimethylated Lys-9 of histone H3 (H3K9me3), HP1α and trimethylated Lys-27 of histone H3 (H3K27me3) are downregulated in cells from Hutchinson-Gilford progeria (HGPS) patients [9]–[11]. Similarly, cells derived from mandibuloacral dysplasia type A (MAD-A) patients exhibit reduction of peripheral heterochromatin, and mislocalization of HP1β, H3K9me3 and lamin B receptor [12]. Defects in laminopathic cells have been attributed to impaired gene expression, lamina misassembly, altered protein-protein interactions and enhanced proteasomal degradation [1]–[5], [13]. Lamin A/C misexpression leads to proteasomal degradation of specific proteins including the retinoblastoma protein (pRb), emerin, nesprin-1α and HP1 [14]–[17]. However, the pathways of proteasomal degradation that are involved are not well understood.

We have earlier shown that expression of laminopathic mutants in HeLa cells leads to proteasomal degradation of specific HP1 isoforms, HP1α and HP1β but not HP1γ, and this is mediated by the F-box substrate adapter protein FBXW10 [17]. HP1α and β are enriched in pericentric heterochromatin and telomeric heterochromatin whereas HP1γ is found in both euchromatin and heterochromatin [18], [19]. Furthermore, we have recently identified three distinct E3 ubiquitin ligase components that are upregulated in lamin A/C knock-down HeLa cells, namely RING finger ubiquitin ligase RNF123, ubiquitin ligase HECW2 and F-box protein FBXW10 [20]. In this study, we have explored the mechanisms underlying the depletion of HP1 isoforms by RNF123 upon lamin A/C knock-down. Our expression analysis, RNAi and binding data suggest that RNF123 mediates the degradation of HP1α and HP1β by directly binding to HP1 through a canonical HP1-binding motif.

## Results

### Downregulation of Endogenous Lamin A/C Leads to Depletion of HP1α and β

**Figure 1:**
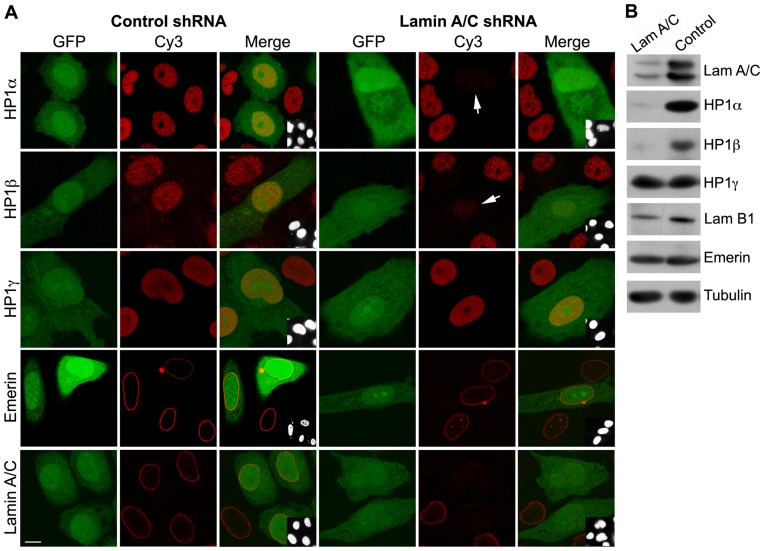
Effect of downregulation of endogenous lamin A/C on chromatin markers. (A) HeLa cells transiently expressing control shRNA or lamin A/C shRNA vectors (co-expressing GFP) were immunostained with the indicated antibodies. Arrows indicate cells showing depletion of HP1α, HP1β or lamin A/C. (B) Western blots of lysates (∼50,000 cells) from stable clones of control shRNA or lamin A/C shRNA (Cl 27) were probed with the indicated antibodies. The results shown are representative of three independent experiments. Bar, 10 µm.

In order to investigate the effects of reduced expression of lamin A/C on HP1 stability, we employed a vector-based shRNA system to knock down endogenous lamin A/C levels as described in Materials and methods. In initial transient transfection experiments, HeLa cells expressing lamin A/C shRNAs and co-expressing GFP were observed to be depleted in HP1α and β but not HP1γ, whereas cells transfected with control shRNA vector did not show any effects on HP1 levels (Fig. 1A). Subsequent experiments were carried out with HeLa cells stably expressing shRNA against lamin A/C transcripts. In one clone that has been described earlier (cl27) [20], there was ∼90% reduction in lamin A/C protein by western blot analysis and nearly complete loss of HP1α and β proteins with no detectable effects on the levels of HP1γ (shown in Fig. 1B). Although the inner nuclear membrane protein emerin is redistributed in cells expressing laminopathic mutants that disrupt the lamina [17], it was not significantly mislocalized to the cytoplasm upon lamin A/C knock-down, suggesting that the small amount of lamin A/C that remains at the nuclear rim in these cells is sufficient for the proper localization of emerin. A previous study has also reported the normal distribution of emerin in lamin A/C depleted cells [21].

In order to determine whether reduction of HP1 α and β was regulated at the protein or transcript level, HP1 transcripts were quantitated by real-time PCR analysis. This data indicated that there were no significant changes in HP1 transcript levels (Table 1), suggesting that lamin A/C knock-down led to degradation of HP1α and β proteins. We further investigated the expression levels of genes bound by HP1 proteins to determine whether there were any changes in expression due to depletion of HP1 isoforms in the lamin A/C knock-down clones. A previous study has identified a large gene family of Kruppel associated box-containing zinc finger (KRAB-ZNF) transcriptional repressors that are the major targets bound by HP1β [22]. To ascertain the effects of depletion of HP1 proteins, the transcriptional status of a few of the ZNF genes located on human chromosome 19 namely, ZNF44, ZNF77, ZNF85, ZNF226 and ZNF266 was determined by real-time PCR analysis of the two clones of HeLa cells stably expressing lamin A/C shRNA. A 2-fold upregulation of ZNF44, ZNF77 and ZNF85 transcripts was observed, but the levels of ZNF226 and ZNF266 were only slightly higher (Table 1). Thus depletion of HP1 proteins in lamin A/C knock-down cells leads to increase in transcripts of certain KRAB-ZNF genes which are normally repressed by HP1β.

**Table 1:** Expression of HP1 and ZNF genes in lamin A/C knock-down clones.

Gene	Cl 26	Cl 27
HP1α	1.88±0.39	1.92±0.22
HP1β	1.19±0.48	1.33±0.22
HP1γ	1.38±0.38	1.58±0.46
ZNF44	2.89±0.65**	2.58±0.54*
ZNF77	2.02±0.28**	2.40±0.58*
ZNF85	2.04±0.24*	2.17±0.67*
ZNF226	1.35±0.42	1.20±0.23
ZNF266	1.63±0.21**	1.56±0.44

Values indicate fold-change in real-time PCR transcripts (upregulation) expressed as mean ± SD calculated from three independent experiments for two stable HeLa clones, Cl 26 and 27 expressing lamin A/C shRNA, compared to a stable clone expressing the control vector.

* *p*≤0.05,

** *p*≤0.01. Cl 26 and 27 showed 2- and 5-fold reduction in lamin A/C transcripts respectively and ∼90% depletion of lamin A/C protein [ref 20].

### RNF123 Mediates HP1 Degradation in Lamin A/C Knockdown Cells

Our earlier studies have shown that RING finger ubiquitin ligase RNF123, HECT-domain ligase HECW2 and the F-box protein FBXW10 are upregulated in HeLa cells expressing laminopathic mutants or lamin A/C shRNA [20]. Further studies have been carried out with RNF123 ubiquitin ligase as it bears a consensus motif for binding to HP1 proteins, as described below.

**Figure 2:**
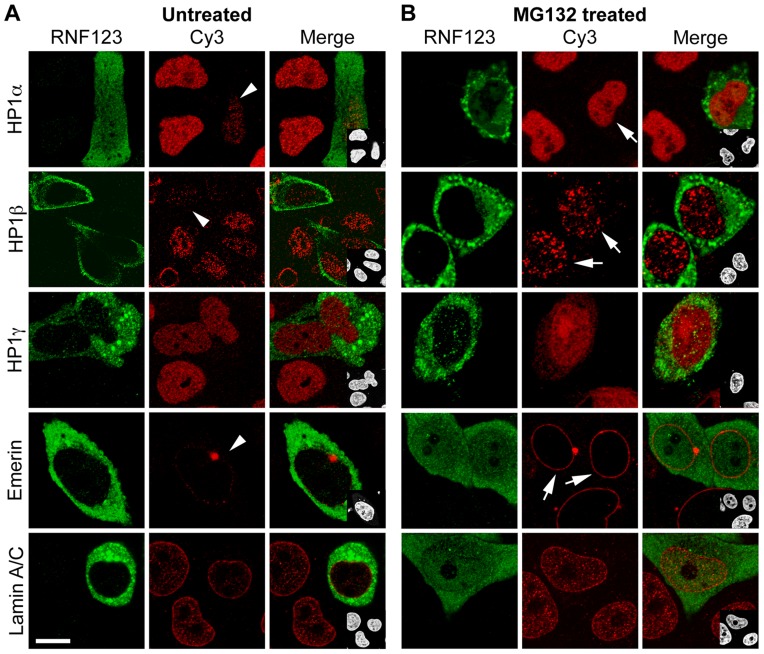
Effects of RNF123 on stability of HP1 proteins. (A) HeLa cells expressing GFP-tagged RNF123 were immunostained with the indicated antibodies and counterstained with DAPI. (B) HeLa cells expressing GFP-tagged RNF123 were treated with 6 µM MG132 and stained. Arrows indicate cells showing depletion of HP1α or HP1β, or dispersal of emerin; arrowheads indicate cells with restored distribution of markers after MG132 treatment. Bar, 10 µm.

**Figure 3:**
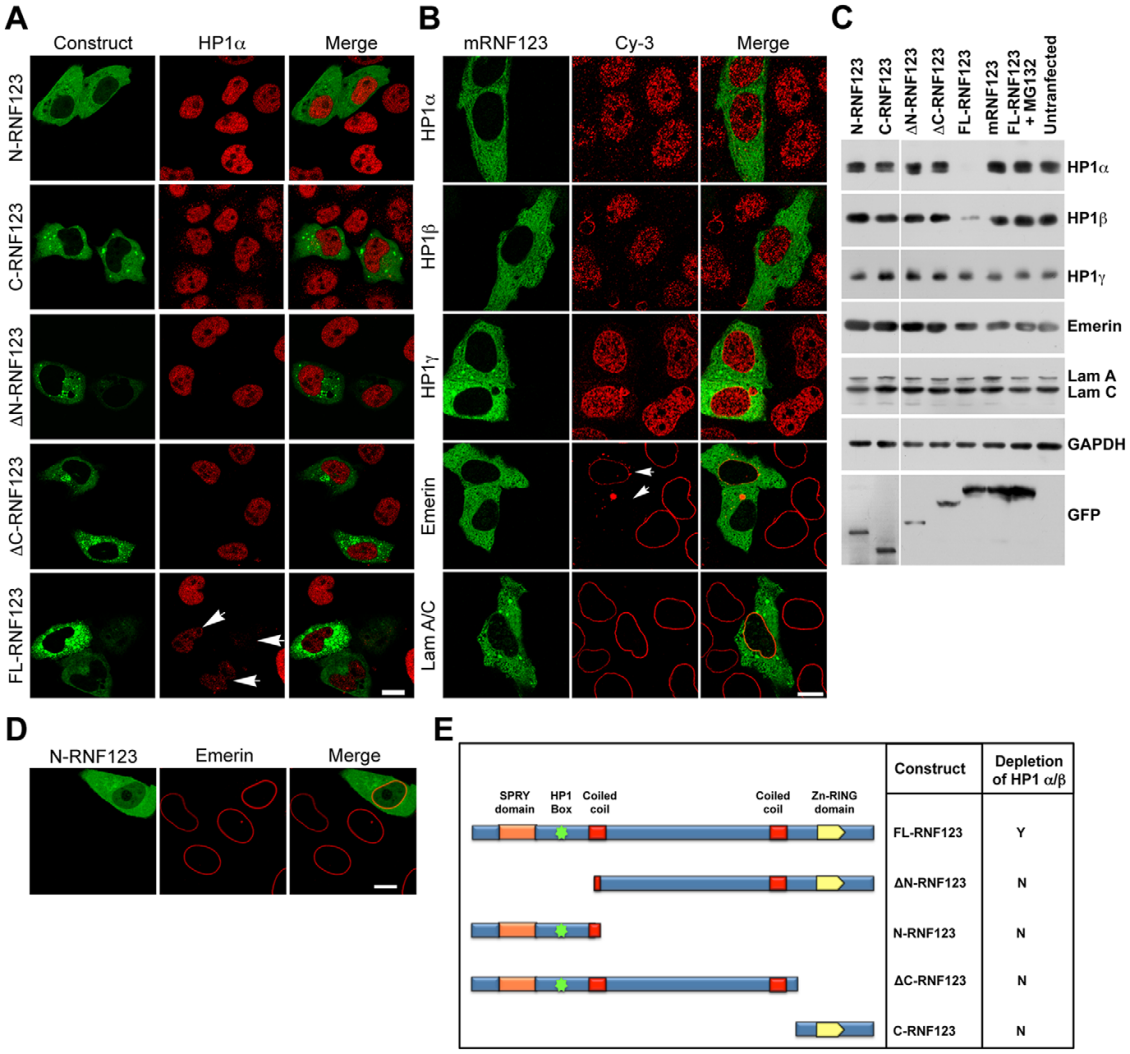
Identification of HP1 binding motif at N-terminus of RNF123. (A) HeLa cells transfected with GFP-tagged deletion constructs were immunostained with antibody to HP1α. Arrows indicate cells showing depletion of HP1α. (B) HeLa cells transfected with GFP-tagged mutant RNF123 (V313D) construct were immunostained with the indicated antibodies. Arrows indicate cells showing dispersal of emerin. All samples were counterstained with DAPI. Bar, 10 µm. (C) Western blots of lysates from cells expressing deletion constructs, wild-type full length RNF123, mutant RNF123 or wild-type full length RNF123 after MG132 treatment. (D) HeLa cells transfected with GFP-tagged N-RNF123 were immunostained with antibody to emerin. (E) Summary of analysis of depletion of HP1α and β with GFP-tagged deletion constructs of RNF123.

RNF123 is a member of the RING family of ligases, one of the largest enzyme families in the human genome that is represented by more than 600 genes [23]. RNF123 (also called KPC1 which stands for Kip1 ubiquitination promoting complex) has previously been shown to degrade cyclin-dependent kinase inhibitor p27^Kip1^ [24]. To examine the role of RNF123 in the stability of HP1, we immunostained HeLa cells ectopically expressing GFP-tagged RNF123 construct with HP1 antibodies. Overexpression of RNF123 caused depletion of HP1α and β, but HP1γ and lamin A/C were not affected (Fig. 2A). Expression of GFP-tagged RNF123 also caused dispersal of emerin into the cytoplasm, as observed earlier upon expression of FBXW10 or laminopathic mutants [17], though emerin levels were not altered. To confirm the role of RNF123 in proteasome-mediated degradation of these proteins, cells expressing GFP-tagged RNF123 were treated with the proteasomal inhibitor MG132. It was observed that proteasomal inhibition by MG132 led to significant restoration of HP1α and β levels as well as emerin relocation to the nuclear periphery (Fig. 2B). Western blot analysis of these samples is shown in Fig. 3. These results indicate that RNF123 ubiquitin ligase mediates the proteasomal degradation of HP1α and β and possibly another protein(s) that is required for the proper localization of emerin. Similar results were obtained with mouse NIH 3T3 cells (data not shown).

### A Canonical HP1 Binding Sequence is Essential for RNF123 Activity

In order to analyze the binding interactions between HP1 proteins and RNF123, we initially created a series of GFP-tagged deletion constructs of RNF123 that lacked specific functional domains. RNF123 has an N-terminal SPRY domain that binds to an associated subunit termed KPC2 and a C-terminal catalytic RING finger domain. The p27-binding region of RNF123 spans beyond its N-terminal SPRY domain and extends towards its C-terminus [25]. The construct ΔN-RNF123 (523–1314 amino acid residues) has a deletion of the SPRY domain and putative substrate binding segment, while the construct ΔC-RNF123 (1–1041 amino acid residues) lacks the RING finger domain. Individual constructs having either SPRY and substrate binding domains (N-RNF123) or only the RING finger domain (C-RNF123) spanning 1–522 and 1042–1314 amino acid residues respectively, were also generated. GFP-tagged full length RNF123 or its deletion constructs were expressed in HeLa cells. The immunofluorescence analysis revealed that deletion of either the SPRY and substrate-binding region or the RING finger domain rendered the molecule ineffective in degrading HP1α (Fig. 3A), indicating that both the substrate-specifying N-terminus and the catalytic RING finger domain are essential for the degradation of HP1 proteins. This was substantiated by western blot analysis with antibodies to HP1 isoforms (Fig. 3C). The expression levels of the constructs were determined by western blot analysis with anti-GFP antibody. The subcellular location of all the deletion constructs was cytoplasmic, as seen with full length RNF123 (Fig. 3A).

The RNF123 motif that binds to HP1 was characterized as follows. Sequence analysis of RNF123 indicated the presence of a canonical HP1 binding sequence PXVXL (X = any amino acid) at position 311–315, downstream of the SPRY domain. This pentapeptide motif is present in many HP1 interacting proteins and non-homologous mutations in this binding motif abolish the protein binding ability of HP1 [26], [27]. The functionality of the HP1 binding motif in RNF123 was determined by mutating the Val residue of PTVLL in RNF123 to Asp residue (V313D). Ectopically expressed GFP-tagged mutant RNF123 was observed to ineffective in depletion of HP1 isoforms as compared to wild-type full length RNF123 by immunofluorescence and western blot analysis, suggesting that the PTVLL pentapeptide motif was involved in recruitment of HP1 proteins to RNF123 followed by their subsequent degradation (Fig. 3B,C). However, this mutation did not reduce the overall catalytic activity of RNF123 as emerin was still dispersed in the cytoplasm in presence of the HP1-binding mutant of RNF123, though its levels were not altered. We confirmed that dispersal of emerin required catalytic activity of RNF123 since N-RNF123 which lacks the catalytic domain, was unable to disperse emerin (Fig. 3D).

**Figure 4:**
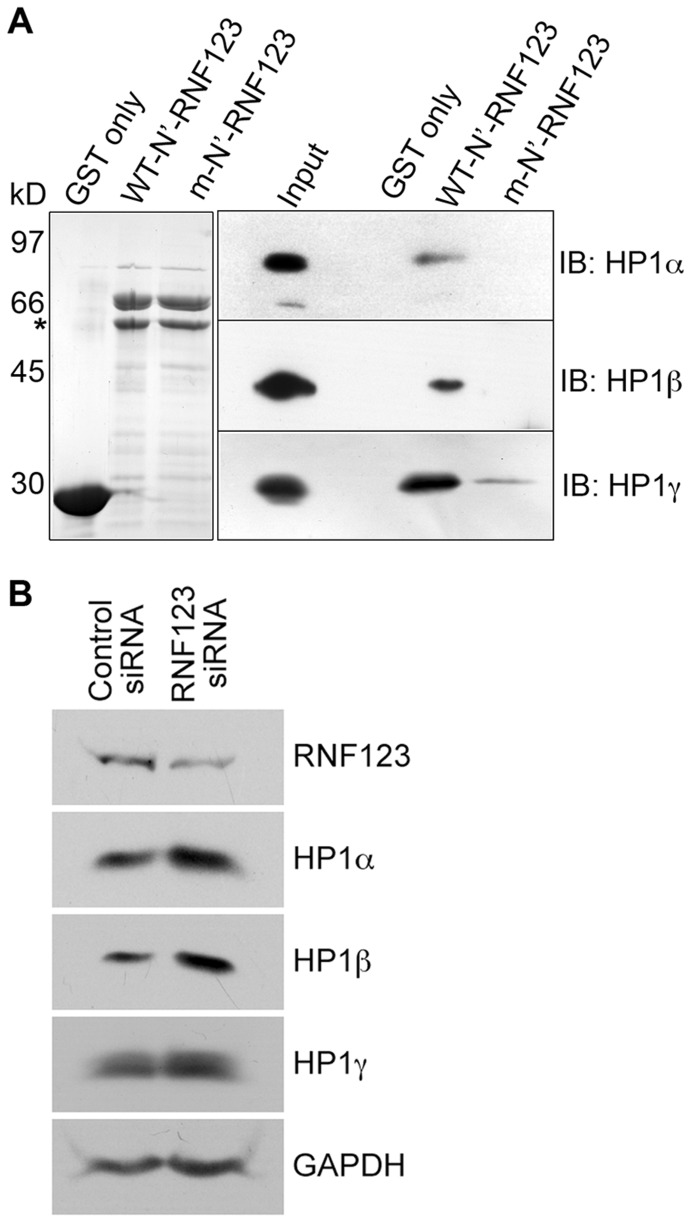
Specificity of RNF123 for HP1 isoforms. (A) Binding studies with N’-RNF123 and HP1 proteins. Left panel: Coomassie blue-stained gel of purified GST, wild-type and mutant GST-N’-RNF123 proteins. Molecular mass markers: phosphorylase b, 97 kD; albumin, 66 kD; ovalbumin, 45 kD; carbonic anhydrase, 30 kD. The asterisk indicates proteolytic degradation products of GST-N’-RNF123. Right panel: Western blots of bound proteins from HeLa lysates probed with antibodies to HP1 proteins. The input lane represents 1% of total lysate from HeLa cells. (B) Effects of RNF123 siRNA treatment. HeLa were transfected with siRNAs for RNF123 or a control siRNA and lysates were probed with the indicated antibodies by western blot analysis.

The association of RNF123 with HP1 via the HP1 binding motif was substantiated by a solid-phase binding assay using recombinant wild-type and mutant (V313D) N-terminal segment of RNF123 (residues 59 to 326) expressed as a fusion with bacterial GST. The GST-N’-RNF123 fusion products migrated at the correct molecular mass as shown in Fig. 4A. HeLa cell lysates were incubated with wild-type or mutant GST-N’-RNF123 bound to glutathione agarose beads and bound proteins were detected by western blot analysis. Wild-type N’-RNF123 bound to all three HP1 isoforms whereas binding to mutant N’-RNF123 was much lower (Fig. 4A). Association of N-RNF123 with HP1 was specific as binding was not observed with GST alone. These experiments establish that RNF123 binds directly to HP1 through the PTVLL binding motif.

In order to validate the role of endogenous RNF123 in HP1 degradation, HeLa cells were transfected with siRNAs to RNF123 as well as control siRNA. Western blot analysis of the lysates showed that RNF123 specific siRNAs reduced endogenous RNF123 levels by ∼60% compared to control siRNA. This led to ∼30% increase in HP1α and ∼60% increase in HP1β but did not affect HP1γ levels (Fig. 4B). The increase in levels of HP1α and β upon RNF123 depletion indicates a role for endogenous RNF123 in HP1α and β degradation.

### HP1β Mobility is Increased in Presence of Lamin Mutants

**Figure 5:**
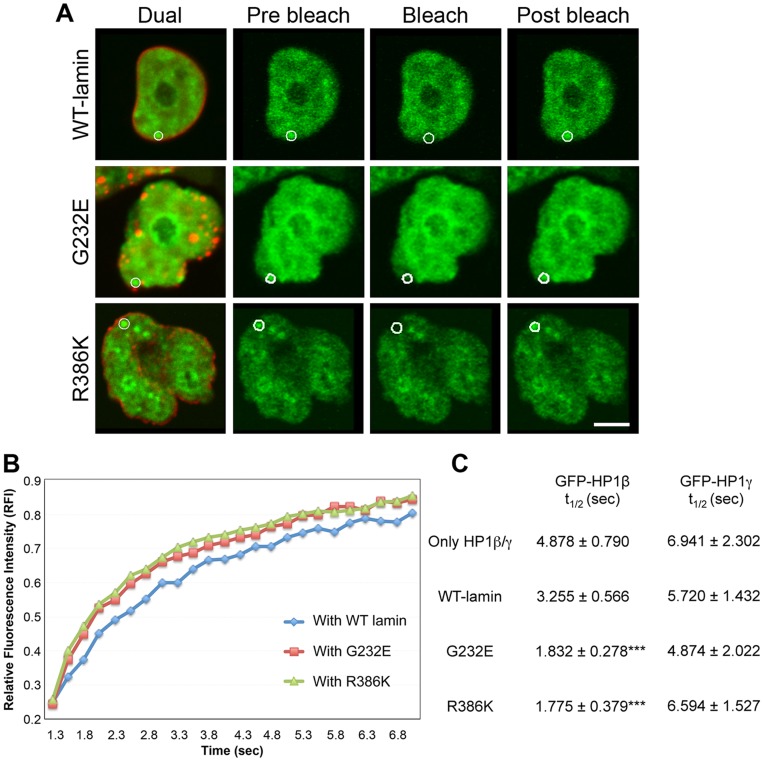
FRAP analysis of GFP-HP1β and GFP-HP1γ. (A) Fluorescence images of typical dual-labeled nucleus (RFP-lamin A and GFP-HP1β) and FRAP processes of GFP-HP1β for the indicated conditions. Circles indicate areas of bleaching where fluorescence recovery was measured. The illustrated postbleach time-point is 40 sec after bleach. Bar, 5 µm. (B) Graphical presentation of fluorescence recovery as change in relative fluorescence intensity (RFI) over time. (C) Comparison of half time of recovery of GFP-HP1 in the background of wild-type and mutant RFP-lamin A. Error bars represent ± sd with n = 25 to 30 individual cells. ***p≤0.001 for mutant cells compared to wild-type lamin A expressing cells.

The above protein expression data and our earlier study [17] indicate that HP1α and β but not HP1γ are specifically targeted for proteasomal degradation upon expression of laminopathic mutations or lamin A/C shRNA in cells. However, our in vitro binding data suggests that all three isoforms bind to the canonical HP1 motif on RNF123. In order to determine whether there were any differences in HP1 dynamics in live cells that might lead to degradation of specific isoforms, we compared the mobilities of HP1β and HP1γ under conditions of lamin misexpression. For this purpose, fluorescence recovery after photobleaching (FRAP) experiments were carried out in HeLa cells co-transfected with GFP-tagged HP1β or HP1γ and an RFP-tagged lamin A construct. A spot of GFP fluorescence in the region of pericentric heterochromatin near the nuclear periphery was bleached and fluorescence recovery was measured. FRAP analysis indicated that there was a significant 2-fold reduction in the half-time of recovery for GFP-tagged HP1β in the presence of RFP-tagged lamin mutants G232E and R386K in comparison with wild-type lamin A, indicating an increase in mobility of HP1β under these conditions (Fig. 5). This difference was not observed with GFP-HP1γ. The half-time of recovery for GFP-tagged HP1β or γ in the presence of wild-type RFP-tagged lamin A was similar to that obtained in the absence of ectopic lamin expression. We infer from this data that the higher mobility of HP1β in the presence of lamin mutants represents displacement from chromatin, and this is not observed with HP1γ.

## Discussion

In the present study, we report that HP1α and β undergo proteasomal degradation in lamin A/C knock-down cells and show by ectopic expression, RNAi and binding studies that the RING finger ubiquitin ligase RNF123 is directly involved in HP1 degradation. Furthermore, GFP-HP1β is destabilized in cells expressing lamin mutants and depletion of HP1β protein in turn causes derepression of KRAB zinc finger transcriptional repressors.

### Effects of Lamin Misexpression on Heterochromatin

The role of the lamina in organization of chromatin has been studied extensively and the deleterious effects of laminopathic mutations or lamin gene disruption on chromatin organization, nuclear morphology and cellular differentiation have been well documented [1]–[5]. In particular, lamin misexpression leads to abnormalities in heterochromatin organization and HP1 localization [6]–[12], [21]. We have previously shown that HP1α and β undergo proteasomal degradation in cells expressing laminopathic mutations and these mutations cause upregulation of transcripts for two specific ubiquitin ligases, RNF123 and HECW2, as well as the F-box protein FBXW10 [17]. We have recently shown that upregulation of these ligase components also occurs in lamin A/C knock-down cells, where they target the DNA damage sensor ATR kinase for degradation [20]. Our findings imply that upregulation of specific ubiquitin ligases is a fairly widespread response to defective lamin assembly due to lamin mutations or knock-down. Our FRAP experiments show that HP1β is more mobile in the presence of lamin mutants, suggesting that it is displaced from chromatin under these conditions. This may lead to its increased proteasomal degradation. This suggestion is consistent with the finding that displacement of topoisomerase II binding protein1 from chromatin results in its increased degradation by a HECT ubiquitin ligase [28].

### RNF123 and HP1 Degradation

The RING finger domain ubiquitin ligases function either as monomeric ligases or as multisubunit ligase complexes. Notably, the monomeric RING ligases have both a substrate binding domain and catalytic RING domain for recruitment of the cognate ubiquitin conjugating enzyme in the same polypeptide, while multisubunit E3 ligases are comprised of substrate specifying factors such as F-box proteins and RING domain proteins that function as a docking platform for ubiquitin conjugating enzymes [23]. RNF123 is the catalytic subunit of the KPC ligase, which is comprised of KPC1 and an associated subunit KPC2, and is required for the degradation of the cyclin-dependent kinase inhibitor p27^Kip1^ during the G0 to G1 transition of the cell cycle [24]. In the present study, we have established a role for RNF123 in the degradation of HP1α and β by ectopic expression studies, RNAi and mutation analysis. We have identified a consensus HP1 binding pentapeptide motif at the N-terminus of RNF123 that upon mutation inhibits the HP1 binding property of RNF123 and prevents HP1 degradation. Ubiquitin ligases involved in HP1 degradation have not been identified earlier; although cullin 4 has been implicated in heterochromatin assembly [29], its levels are not altered in laminopathic cells [17]. Our earlier study has shown that HP1 is targeted by FBXW10 [17] and our initial experiments suggest that HECW2 also degrades HP1α and β (data not shown). Degradation of HP1 by multiple ligases is not unusual since key regulatory proteins such as p27^Kip1^ and ATR kinase are targeted by several ubiquitin ligases [20], [24], [30].

The loss of HP1 isoforms can have severe effects on chromatin function and gene regulation, leading to lethality in *Drosophila* and mouse [31], [32]. Since HP1β has been reported to modulate the activity of KRAB-ZNF genes [22], we investigated the effects of HP1 depletion on the transcript levels of some of these genes. We observed that depletion of HP1 proteins due to knock-down of A-type lamins significantly upregulated certain KRAB-ZNF transcripts. This implies that lamin misexpression may lead to dysregulation of downstream target genes due to degradation of chromatin proteins like HP1α and β.

### Proteasomal Degradation of Lamin-associated Proteins

Laminopathic mutations or downregulation of lamin A/C leads to degradation of a number of key regulatory proteins that bind directly or indirectly via chromatin to lamin A/C. Lamin A/C knock-down human osteosarcoma cells and embryonic fibroblasts derived from lamin A/C knock-out mice display enhanced proteasomal degradation of pRb [14]. The homozygous nonsense mutation Y259X in *LMNA* gene has been reported to cause mislocalization and subsequent proteasomal degradation of emerin and nesprin-1α [15], although the ubiquitin ligases involved have not been identified in both these studies. We have proposed that the aberrant assembly of the lamina upon lamin misexpression is likely to lead to disruption of normal interactions between lamins and chromatin proteins or lamin-binding proteins, leading to release of bound proteins that are subsequently targeted for degradation by their cognate ligases. An increase in the engagement or turnover of these ligases in the nucleus may then activate their increased expression via a feedback loop [20]. Our present data on the displacement of HP1β from chromatin in cells expressing laminopathic mutations is consistent with this model. Hence inappropriate release of bound proteins due to lamina disorganization may be a general mechanism leading to their eventual degradation.

## Materials and Methods

### Plasmid Constructs

Wild-type GFP-lamin A, RFP-lamin A and mutant lamin mammalian expression constructs have been described earlier [17], [33], [34]. For shRNA-mediated knockdown of endogenous lamin A/C, pcDNA6.2-GW/EmGFP-miR LMNA and control vector (Life Technologies) were employed. The shRNA targeted lamin A/C mRNA at nucleotide positions 849–869, corresponding to the sense sequence of GAAGGAGGAACTGGACTTCCA. The control vector harboured the scrambled sequence GAAATGTACTGCGCGTGGAGAC, which does not target any known vertebrate gene. GFP-HP1β and HP1γ expression vectors were generously provided by Peter Hemmerich (Institute of Molecular Technology, Jena, Germany). An RNF123 (KPC1) plasmid construct was kindly provided by Bo Cen (Medical University of South Carolina, USA). GFP-tagged constructs of RNF123 were made as follows. Full-length RNF123 was subcloned into XhoI-SmaI digested pEGFP-C1 mammalian expression vector (Clontech). The N-RNF123 construct was made by ligating the HindIII-digested N-terminal fragment (1.6 kb) of RNF123 to pEGFP-C3, while the N-terminal deletion mutant of RNF123 (ΔN-RNF123) was made by subcloning the 2.6 kb HindIII-SmaI fragment of RNF123 into pEGFP-C1. An EcoRI-SmaI fragment (900 bp) from the C-terminus of RNF123 which contained only the RING finger domain was cloned into pEGFP-C2. The C-terminal deletion construct (ΔC-RNF123) that does not harbour the RING finger domain was cloned into XhoI-EcoRI digested pEGFP-C1. The RNF123 mutant (V313D) was generated by mutating Val-313 to Asp-313 using the QuikChange™ Site-Directed Mutagenesis kit (Stratagene Cloning System, La Jolla, CA) according to the manufacturer’s instructions using a combination of mutagenic primers 5′GCGGGGCCAGCCCACCGACCTCCTCACACTGGCCC3′ and 5′GGGCCAGTGTGAGGAGGTCGGTGGGCTGGCCCCGC 3′. N-terminal fragments spanning amino acid residues 59 to 326 of both wild-type and mutant RNF123 were amplified by PCR (primers given in Table S1) and cloned into the bacterial expression vector pGEX-5X3.

### Cell Culture, Generation of Stable Cell Lines, RNAi and Drug Treatment

HeLa cells were grown in DMEM supplemented with 10% FBS at 37°C in a humidified atmosphere containing 5% CO_2_ [17]. Plasmid constructs were transiently transfected into HeLa cells for 24 h using Lipofectamine 2000 (Invitrogen) by standard protocols. For treatment with proteasomal inhibitors, cells were incubated with 6 µM MG132 (Calbiochem) for 18 h starting at 6 h after transfection of plasmids. Stable lines expressing control or lamin A/C shRNA plasmids were generated by blasticidin selection as described [20]. For downregulation of RNF123 using RNAi, RNF123 mRNA was targeted using a pool of siRNAs (Bioserve Technology, Hyderabad, India) having the sequences 5′UCAACAGCGUCCUCAAUCA3′, 5′CCACCAUCGUGUCUGUAGA3′ and 5′GUGGCCAUCAACAGCUACAGU3′ corresponding to positions 3215–3233, 4004–4022 and 2731–2751 respectively. The sequence 5′UUCUCCGAACGUGUCACGU3′, predicted not to target any known mammalian mRNA sequence, was used as a negative control. A 10 nM solution of siRNAs was transiently transfected into HeLa cells for 36 h using Lipofectamine 2000.

### RNA Isolation and Quantitative Real-time PCR Analysis

Total RNA was extracted from HeLa cells stably expressing the shRNA constructs against lamin A/C or control vector and 1 µg of RNA was reverse transcribed using Superscript II reverse transcriptase kit (Invitrogen) as per the manufacturer’s instructions. Amplification of PCR products was quantitated using SyBR green dye (ABI) and monitored on an ABI prism 7900 HT sequence detection system. Melting curve analysis was done for each amplicon. The ΔΔCt method was used for quantitation using HPRT1 (hypoxanthine phosphoribosyl transferase 1) as endogenous control. The analysis for each gene was done in triplicate and three independent biological replicates were carried out. Gene expression in lamin A/C knock-down stable clones was expressed as fold change in comparison with HeLa cells stably expressing control shRNA vector. The gene specific primers used for real-time PCR analysis are given in Table S1.

### Antibodies and Western Blot Analysis

A polyclonal antibody to lamin A/C and a mouse mAb to tubulin were from Santa Cruz Biotechnology. Mouse mAbs to HP1α, β and γ were obtained from Millipore. Polyclonal antibodies to GFP, GAPDH and HP1α, β and γ were from Abcam. A mouse mAb to emerin (clone 4G5) was obtained from Novocastra Laboratories and a mouse mAb to RNF123 was from Sigma-Aldrich Corporation. For western blot analysis, cells were lysed in Laemmli’s sample buffer, boiled and electrophoresed through SDS-10% polyacrylamide gels. Gels were electroblotted onto PVDF membrane filters and blocked for 1 h at ambient temperature in 5% BLOTTO in Tris-buffered saline containing 0.1% Tween-20. Blots were incubated with primary antibody for 2 h, followed by peroxidase conjugated-secondary antibody in Tris-buffered saline containing 0.1% Tween-20 for 1 h. Antibody binding was analyzed using a chemiluminescence kit from Roche Applied Science and scanned blots were quantitated with ImageJ software.

### Solid-phase Binding Assays

Recombinant GST-N’-RNF123 clones and pGEX-5X3 vector were expressed in *E. coli* BL21(DE3)-pLys(S) by induction with 1 mM IPTG for 3 h at 37°C. Cells were lysed in 100 mM EDTA, 1% Triton X-100, 10 mM sodium phosphate buffer, pH 7.2, 150 mM NaCl, 1 mM PMSF, 0.5 mM DTT and 2% protease inhibitors. The supernatants containing the expressed proteins were incubated with glutathione agarose beads for 2–3 h at 4°C, centrifuged and washed with lysis buffer. HeLa cells were lysed in buffer containing 50 mM Tris-Cl pH 7.5, 150 mM NaCl, 10% glycerol, 0.5% NP-40, 2 mM EDTA, 2 mM EGTA, 2 mM PMSF, 2 mM NaF, 2 mM Na_3_VO_4_ and protease inhibitors, and the supernatant was pre-cleared with glutathione agarose beads. The pre-cleared supernatant was incubated with equal quantities of beads bound to either GST or GST-fusion proteins for 12 h at 4°C, centrifuged and washed with lysis buffer, separated by SDS-PAGE and analyzed by western blotting.

### Immunofluorescence Microscopy

HeLa cells were fixed with 4% formaldehyde in phosphate-buffered saline (PBS) for 10 min followed by treatment with 0.5% (v/v) Triton X-100 for 6 min at room temperature. Cells were subsequently incubated with 0.5% gelatin in PBS for 1 h and then incubated with primary antibody for 1 h followed by Cy3-conjugated secondary antibody for 1 h. Samples were mounted in Vectashield (Vector Laboratories) containing 1 µg/ml DAPI. Fluorescence microscopy of the immunostained cells was performed using a LSM510 META/NLO confocal microscope. Images were analyzed with LSM510 META software and assembled using Photoshop CS3.

### Live Cell Microscopy

HeLa cells transiently co-transfected with RFP-tagged lamin A constructs and GFP-tagged HP1β or HP1γ [35] were seeded on a LabTekII chamber slide and grown for 24 h. Fluorescence recovery after photobleaching (FRAP) experiments were performed on an LSM510 META/NLO inverted confocal microscope with a 63x/1.20 Wkorr planapochromat water objective lens as described [36]. A GFP-HP1 spot of 1 µm in radius was bleached for 0.33 sec using the 900 nm laser line at 80% laser power, and a series of images was acquired at 304-msec intervals immediately after bleaching. RFP was not bleached under these conditions. Recovery of fluorescence was observed for 50 sec. All intensities were background subtracted for calculation of FRAP curves, which represented an average of 25–30 individual cells bleached under identical conditions in at least three independent experiments. The relative fluorescence intensity (RFI) at each time point was calculated as described by Phair and Misteli [37], using the equation RFI = (It/Tt)/(I_0_/T_0_) where I_0_ and It are the average intensity of the region of interest before and after bleaching at time t, and T_0_ and Tt are the total intensity in the nucleus before and after bleaching at time t. Non-linear curve fitting was carried out using MS-Excel. In control experiments in which formaldehyde-fixed cells were analyzed, no recovery of fluorescence was observed.

## Supporting Information

Table S1 **List of PCR primers.**
(DOC)
