# Supplementary material for: Ubiquitin ligase RNF123 Mediates Degradation of Heterochromatin Protein 1α and β in Lamin A/C Knock-Down Cells
Source: PLoS One. 2012 Oct 15;7(10):e47558. doi: 10.1371/journal.pone.0047558 (PMC3471868; doi:10.1371/journal.pone.0047558)
Supplement: Table S1 — List of PCR primers. (DOC) [file pone.0047558.s001.doc]

**Table 1S List of PCR primers**

|  | Gene | Forward primer 5’ to 3’ | Reverse primer 5’ to 3’ |
| --- | --- | --- | --- |
| 1 | HP1α | GAAAAACTTGGATTGCCCTGAG | GCGATATCATTGCTCTGCTCTCTC |
| 2 | HP1β | CCGTCGAGTGGTAAAGGGCAAAG | GCGCTTGCCTCCCTCTGATTTATC |
| 3 | HP1γ | GCTGGCAAAGAAAAAGATGGTAC | CATCAATTCTCCACTGCTGTCTGT |
| 4 | HPRT1 | GCCCTGGCGTCGTGATTA | CATCTCGAGCAAGACGTTCAGT |
| 5 | ZNF44 | CTAGAGCGAGACTCCATCTCAA | GCTGGCATCTGTGGATTTAGG |
| 6 | ZNF77 | CAGAGGGACGTTTTTGGGAAT | GTGGGATTTGGTGCTGATCTC |
| 7 | ZNF85 | TCATTTTGCCCAAGACCTTTG | CCTCCTTTGTGCATCTTACACTCA |
| 8 | ZNF226 | CCAGAGGAAGCTGTACCGAGAT | TGCTGTCGTCATTATCCAAAGC |
| 9 | ZNF266 | GTGGAGAAGACCCTGCATCAG | TGTGGGATTCAGAAAGGTATTCC |
| 10 | N’-RNF123 | cgggatcccgCTGAACTTCCAGAACCTGCCAGAACA | cggaattccgGCGGTGCAAAGTGATGGAAGATGTG |
